# The Life and Legacy of Dr. Jonathan Letterman (1824–1872): The Father of Modern Battlefield Medicine

**DOI:** 10.7759/cureus.102779

**Published:** 2026-02-01

**Authors:** Jack Carter, Nicole Meyers

**Affiliations:** 1 Medicine, Lake Erie College of Osteopathic Medicine, Bradenton, USA; 2 Osteopathic Manipulative Medicine, Lake Erie College of Osteopathic Medicine, Bradenton, USA

**Keywords:** battlefield medicine, combat, historical vignette, medical innovation, medical stories, military healthcare, military history

## Abstract

Dr. Jonathan Letterman was a crucial innovator in the fields of combat and emergency medicine during the American Civil War. In a time that saw some of the bloodiest battles being fought on American soil, Dr. Letterman provided essential breakthroughs in military medicine that prevented thousands of soldiers from perishing. Through improvements in military camp sanitation, the establishment of an ambulance corps and the creation of an official evacuation plan and triage system for the wounded, Dr. Letterman’s work not only helped save the lives of countless soldiers fighting in the Civil War, but in future wars to come as well. In addition, his service to his men and his country helped champion mandatory healthcare for the wounded soldier, an idea that would be eventually passed into Congressional law as the Letterman Plan. His life and legacy of improving military medicine amidst America’s deadliest war are a testament to how he is aptly nicknamed “The Father of Battlefield Medicine”.

## Introduction and background

**Figure 1:**
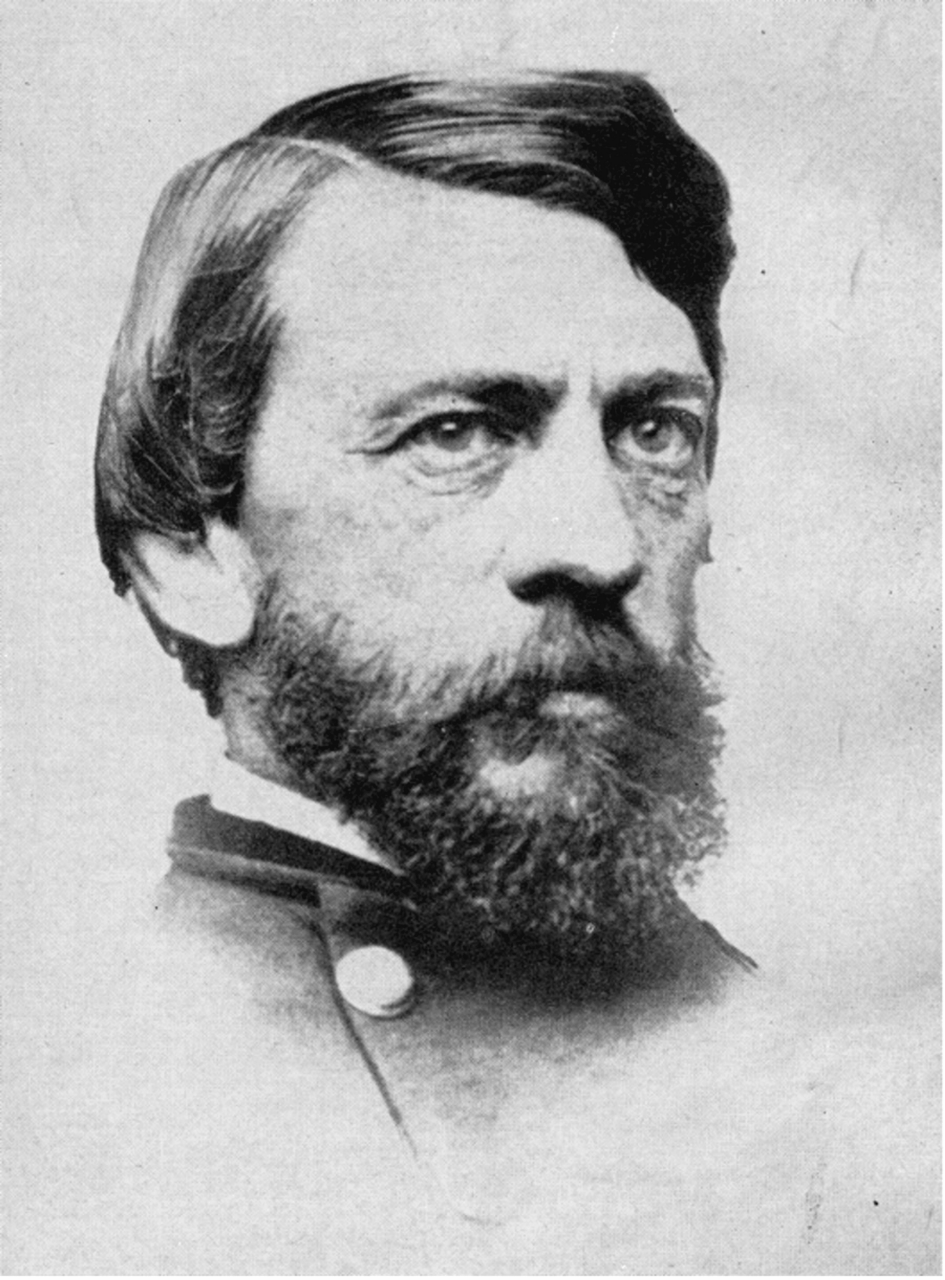
Portrait of Major Jonathan Letterman Image source: reproduced with permission from the National Museum of Civil War Medicine [1]

Dr. Jonathan Letterman, an unsung Union Army surgeon and the medical director of the Army of the Potomac, was a pioneer in the fields of emergency and combat medicine. Using the horrors of the American Civil War to pioneer an innovative restructuring of the Union Army’s Medical Corps, he dedicated himself to improving emergent care for wounded soldiers left to die on the battlefield during one of the deadliest wars in American history. This article recounts his life, the breakthroughs he made during the Civil War, and his lasting legacy in American military medicine (Figure 1).

## Review

Early life

Jonathan Letterman was born on December 11, 1824, in Canonsburg, Pennsylvania, into an affluent family. His father was a well-known surgeon in Western Pennsylvania, and his mother was the daughter of a successful businessman. As a result, both Jonathan and his younger brother William received private tutoring during their early education [2]. In 1842, Jonathan enrolled at Jefferson College in Canonsburg, completing his studies and graduating in 1845. Inspired by his father’s medical career, he went on to attend Jefferson Medical College in Philadelphia, where he earned his medical degree in 1849.

Early military service

After completing medical school, Dr. Letterman joined the U.S. Army Medical Services as an assistant surgeon in 1849. He initially served in Florida during campaigns against the Seminole Indians, then moved to Fort Ripley, Minnesota. Later, he marched with troops from Fort Leavenworth, Kansas, to Fort Defiance, New Mexico, where he assisted Colonel William W. Loring in operations against the Gila Apache [3]. He remained stationed in New Mexico until a granted leave of absence in 1858. In 1859, he was sent to Fort Monroe, Virginia, for six months and then began working under Colonel Richard S. Satterlee, the Chief Medical Purveyor for the US Army in New York. In December of 1859, he was sent to California to assist in Major James H. Carleton’s expedition against the Ute Indians [4]. For the first 10 years of his military service, Dr. Letterman remained on the fringes of the American frontier, treating troops far away from established hospitals, using his improvisations and ingenuity to treat and transport patients in urgent situations, a skill that would certainly be used and tested in the coming years of his life [3].

American Civil War

**Figure 2:**
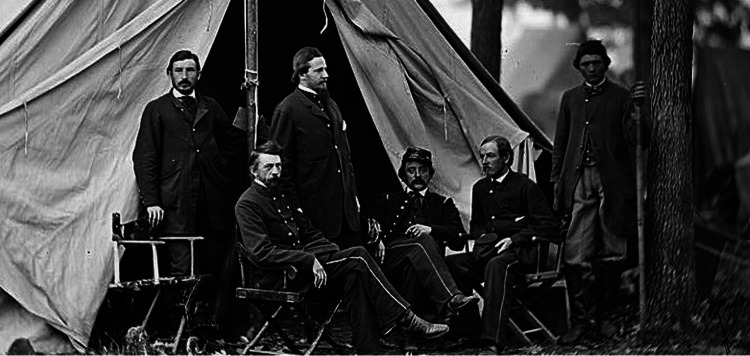
Dr. Letterman, seated furthest left and medical staff. Image source: reproduced with permission from the Library of Congress [7]

On April 12, 1861, forces from the Confederate States of America unleashed an artillery barrage upon Union forces at Fort Sumter, South Carolina, officially beginning the American Civil War. In July of that same year, the Union Army retaliated, advancing south toward the Confederate capital of Richmond, Virginia, with the intent of capturing it. What resulted was the First Battle of Bull Run at Manassas Junction, Virginia - a battle that saw over 460 Union soldiers killed and another 1,124 wounded, many of whom succumbed to their injuries on the battlefield, due to there being little to no ambulance service to retrieve them [1]. In the meantime, Dr. Letterman was recalled from California to assist Major General George B. McClellan’s Army of the Potomac (the Union Army’s primary fighting force in the Eastern Theatre of the American Civil War) in November of 1861. In May 1862, Letterman was appointed medical director of West Virginia, and just a month later, he received a presidential appointment as the medical director of the Army of the Potomac, placing him in charge of medical operations for one of the Union’s most important armies (Figure 2) [4]. His promotion was just in time. Upon returning from the White House, Dr. Letterman found McClellan’s Army in pieces after the Seven Days Battles. In seven days, seven battles had commenced just north of Richmond, ultimately resulting in a Confederate victory and the deaths of nearly 1,734 Union soldiers, plus an additional 14,000 wounded - the second bloodiest battle of the entire Civil War [5]. With such a large number of wounded men, McClellan’s medical corps was overwhelmed. Supplies had nearly been exhausted, hospital tents lay abandoned or destroyed, and medical officers were severely lacking in number [2]. Prior to the war, most promotions in the US Army Medical Corps had been based on seniority and not merit. Because of this, over half of its frustrated physicians resigned and joined the South in hopes of better pay once war broke out [2,6]. Upon seeing its dire state, Letterman quickly realized the need for an immediate reorganization and restructuring of the medical corps within the Union Army and got to work making groundbreaking improvements.

Improvements in camp sanitation and hygiene

One of Dr. Letterman’s first initiatives was to improve camp sanitation and overall hygiene. He organized the relocation of large numbers of sick, injured, and exhausted soldiers to safer areas, away from healthy troops and the chaos of the front lines [4]. Letterman implemented wide-ranging improvements in camp life, focusing on sanitation, food, and living conditions. He ensured proper disposal of waste, oversaw the cooking and cleaning of meals, and provided soldiers with larger, more nutritious rations, including a guaranteed breakfast. Barracks were rebuilt to offer better sleeping quarters, and clean uniforms became mandatory. These measures gradually lifted morale, and within less than a month of Letterman assuming his role as chief medical officer, the disease rate in the army fell by one-third, demonstrating the immediate effectiveness of his reforms [6]. This earned Letterman praise from Major General McClellan himself, who stated that “all the remarkable energy and ability of Surgeon Letterman were required to restore the efficiency of his department … the health of the Army was vastly improved by the sanitary measures which were enforced at his suggestion” [2].

Development of an ambulance corps

**Figure 3:**
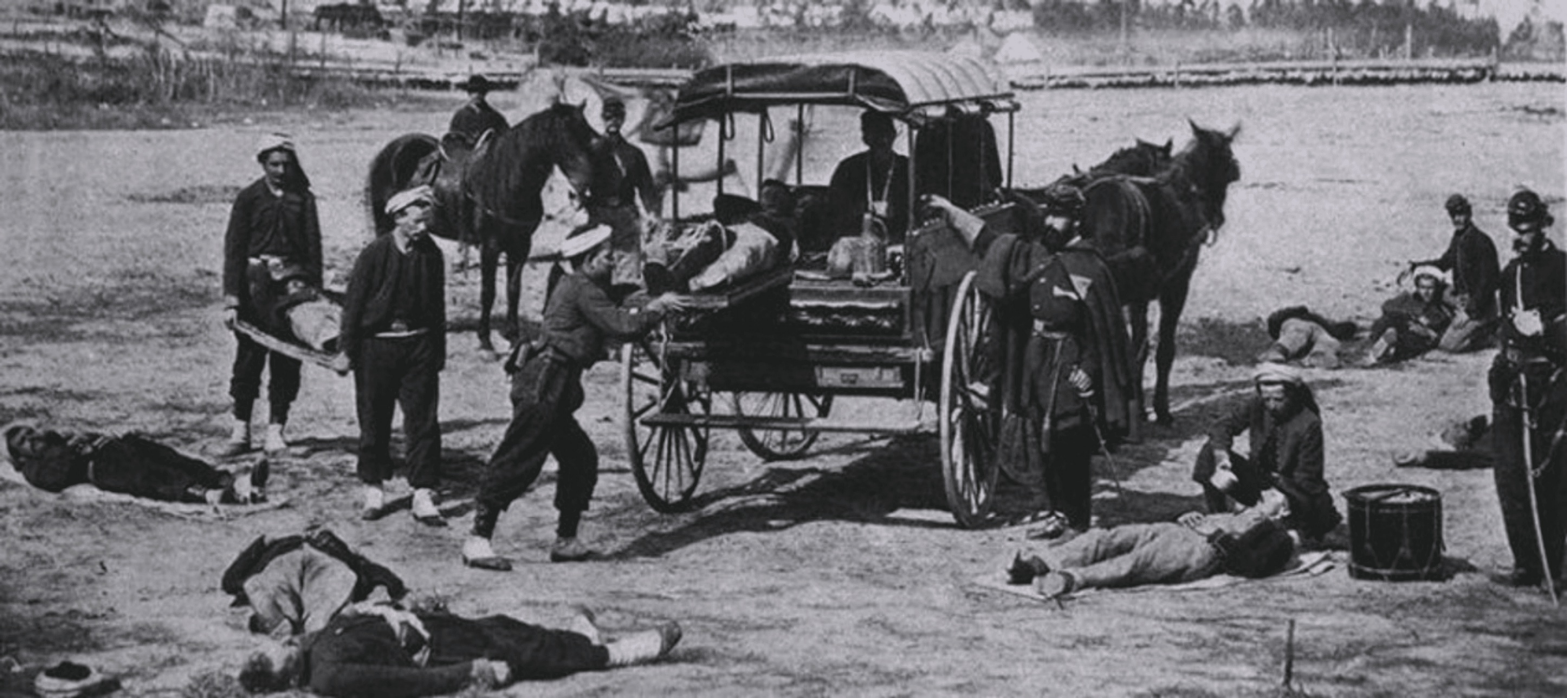
Ambulance drill in the field in preparation for the Battle of Antietam in 1862 Image source: reproduced with permission from the Digital Collections - National Library of Medicine [11]

Prior to Letterman’s reforms, wounded soldiers were frequently left on the battlefield with little hope of timely aid. Unless a fellow soldier carried them away or they managed to return on their own, they could remain alone for days, enduring injuries, exposure, hunger, and thirst. For example, after the Second Battle of Manassas in 1862, it took more than a week to evacuate the wounded [8]. Regimental musicians with no medical training were often given the task of hauling the wounded away and most of them carried off more supplies on their stretchers than men [9]. Seeing this, Dr. Letterman established the first US Ambulance Corps, making the finding and transporting of wounded soldiers their sole responsibility (Figure 3) [9]. Letterman designated specific men to serve as permanent ambulance drivers and implemented a standardized training program to ensure their competency [2]. He structured the Ambulance Corps in a way that mirrored the organization of the army. Each infantry corps had an ambulance team led by a captain, while lieutenants oversaw the ambulances within smaller divisions, and sergeants managed those assigned to individual regiments. This hierarchical system clarified leadership within the ambulance units and enabled physicians to concentrate on caring for the injured [10]. Letterman’s new ambulance system was so effective for McClellan’s Army of the Potomac that Congress institutionalized it for all Union Army divisions in 1864 [9].

Institution of an evacuation plan and triage system

After building an ambulance corps, Dr. Letterman next set out to restructure the evacuation of the wounded and installed a triage system that is still used to this day. His evacuation plan consisted of a three-tiered system consisting of three stations. First were the field dressing stations, which were located on or next to the battlefield, where medical personnel could immediately apply initial dressings and tourniquets to wounds. Second were the field hospitals, located a bit farther away from combat, usually in a home or barn, which served as designated sites for emergency surgery and additional treatments. Lastly, the large hospitals were far away from the battlefield, usually in a city, and allowed for longer- term treatment [8]. All these measures allowed for the efficient removal and treatment of wounded men in the chaos of battle and became the basis for military medical administration for the Union Army for the rest of the Civil War [9]. This new evacuation system naturally allowed for a new triage system and promoted graduated echelons of care as well. At field dressing stations, medical personnel provided initial care, applying tourniquets, administering morphine, and offering water, while quickly assessing each soldier’s condition. Those with severe injuries were transported by the ambulance corps to field hospitals for surgery or amputations, ensuring they received specialized care. Meanwhile, soldiers with minor wounds stayed closer to the front lines, allowing them to recover and return to duty swiftly. Letterman’s system not only improved survival rates but also streamlined battlefield medical care, keeping the army stronger and more effective [6]. Dr. Letterman’s implementation of these two systems led to remarkable results - the Union Army’s mortality rate from battle wounds dropped significantly, falling from 25.6% in the first year of the Civil War to 13.3% following his arrival in 1862 [10].

Camp Letterman

**Figure 4:**
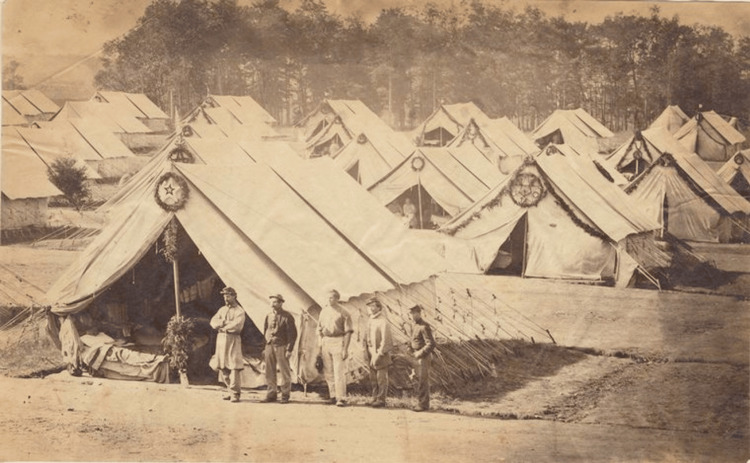
Camp Letterman near Gettysburg in 1864 Image source: reproduced with permission from the Manuscripts and Archives Division, The New York Public Library [12]

Dr. Letterman’s work could not have come at a better time, as the major battles of Antietam (12,401 Union casualties), Fredericksburg (12,653 Union casualties), and eventually Gettysburg (23,049 Union casualties) were soon to be fought. During the Battle of Fredericksburg, Union Surgeon George Stevens wrote that “the medical department has become so thoroughly systematized that wounded and sick men were cared for better than they had ever been in any army before … by the efficient and earnest medical director of the army, Dr. Letterman; to whom belongs the honor of bringing about this most desirable change” [6]. At Gettysburg, the bloodiest battle in the Civil War, Dr. Letterman established a fully functioning general hospital surrounded by smaller field hospitals using improvised churches, barns, and townhomes. The site, known as “Camp Letterman,” featured numerous tents, each accommodating up to 40 cots equipped with mattresses and sheets - a rare comfort for many wounded soldiers who were accustomed to receiving treatment while lying on the ground. It became a symbol for how far medical intervention had come from the beginning of the war and gave many soldiers hope amidst the most horrific battles being fought (Figure 4) [2].

The Letterman Plan and his legacy

Letterman would continue to refine the Army’s system of medical care, dedicated to improving the chance of survival for its soldiers. These changes, known as the Letterman Plan, ensured that a wounded soldier would be cared for from the moment they were injured all the way through to their long-term care. Dr. Letterman resigned as medical director of the Army of the Potomac in January 1864, but his innovative system of battlefield medical care endured. Just two months later, in March, Congress formally enacted his plan into law, ensuring that his reforms were implemented throughout all Union armies and would continue to shape military medicine long after the Civil War [10,13]. After his resignation, Letterman moved to San Francisco, where he served two terms as city coroner. In 1866, he published his memoirs, Medical Recollections of the Army of the Potomac, in which he detailed his experiences as the Army’s medical director. The work not only documented the challenges and innovations of wartime medicine but also cemented his lasting influence on military medical practices [8,9]. Dr. Jonathan Letterman passed away in 1872 from a chronic intestinal illness and was buried at Arlington National Cemetery in Virginia. His gravestone venerates him as the individual “who brought order and efficiency into the medical service and who was the originator of modern methods of medical organization in armies,” a testament to the enduring impact of his innovations on military medicine [8]. On November 13, 1911, the Army Hospital at the Presidio of San Francisco was officially named Letterman Army Hospital to honor Dr. Jonathan Letterman’s contributions. A small commemorative tablet along York Road by Gettysburg also recognizes him and the “Camp Letterman General Hospital,” highlighting his pioneering work in organizing battlefield medical care during the Civil War [2].

## Conclusions

Dr. Jonathan Letterman’s passion for the injured soldier drove him to make outstanding breakthroughs in the fields of battlefield and emergency medicine. Even amidst some of the darkest and bloodiest days in United States history, Letterman’s commitment to saving lives gave his country hope in a perilous time. His ambulance corps was the first of its kind in military medicine and proved to be an effective solution toward his goal of refining care for those maimed in battle. His evacuation system for the wounded has been used with masterful success in countless wars since the American Civil War, saving thousands if not millions of lives and his triage policies are implemented across emergency departments worldwide. His official plan of treating a patient all the way throughout their recovery became immortalized in Congressional law and serves as a testament to his vision of providing the best care for his soldiers. Because of his pioneering innovations in the field of military medicine and his devotion to the health of the men who served under his watch, Dr. Jonathan Letterman thoroughly earned the distinguishment as the Father of Battlefield Medicine.

## References

[1] Price Price, David David (2025). Major Jonathan Letterman - civil war medical innovator. Major Jonathan Letterman - Civil War Medical Innovator.” National Museum of Civil War Medicine, 2 Feb.

[2] L’éinelle F (2025). Jonathan Letterman. Jonathan Letterman.

[3] Phalen J (1947). The life of Jonathan Letterman. Records of the American Catholic Historical Society of Philadelphia.

[4] Clements B (1883). Memoir of Jonathan Letterman. J Mil Serv Inst.

[5] Miller Miller, William William (2025). The seven days battles. American Battlefield Trust, American Battlefield Trust, 4 Feb.

[6] (2025). Techniques of Civil War medical innovator Jonathan Letterman still used today. https://www.army.mil/article/216935/techniques_of_civil_war_medical_innovator_jonathan_letterman_still_used_today.

[7] Gardner Gardner (2025). Dr. Jonathan Letterman, medical director of the army of the potomac and staff. Dr. Jonathan Letterman, medical director of the Army of.

[8] Jonathan Jonathan (2025). Jonathan Letterman. http://www.battlefields.org/learn/biographies/jonathan-letterman.

[9] Letterman Letterman, Jonathan.” Letterman, Jonathan - UAB Libraries, Arnold G (2025). Letterman, Jonathan. https://library.uab.edu/locations/reynolds/collections/civil-war/medical-figures/jonathan-letterman.

[10] Labbe Labbe, Savannah. “John (2025). A complete transformation of medicine: John Letterman's ambulance corps. Gettysburg College, 9 Jan.

[11] (2025). U.S. Civil War: Medical and sanitary affairs. U.S. National Library of Medicine, National Institutes of Health.

[12] (2025). Battlefield of Gettysburgh: U.S. general hospital. https://digitalcollections.nypl.org/items/510d47dd-e803-a3d9-e040-e00a18064a99.

[13] Moses Moses, Rachel Rachel (2025). Doctor's orders: Jonathan Letterman. Doctor’s Orders - Jonathan Letterman.” NMCWM-Lesson-Plan-Letterman, National Museum of Civil War Medicine, 11 May.

